# Characterisation of aphid antixenosis in aphid‐resistant ancestor wheat, *Triticum monococcum*

**DOI:** 10.1002/ps.8380

**Published:** 2024-08-17

**Authors:** Alexander N. Borg, József Vuts, John C. Caulfield, David M. Withall, M. John Foulkes, Michael A Birkett

**Affiliations:** ^1^ Protecting Crops and the Environment, Rothamsted Research Harpenden UK; ^2^ Division of Plant and Crop Sciences The University of Nottingham Loughborough UK

**Keywords:** aphid resistance, *Triticum monococcum*, wheat, *Sitobion avenae*, volatile organic compounds, antixenosis

## Abstract

**BACKGROUND:**

Due to the increasing presence of insecticide resistance across cereal aphid populations, new aphid management strategies, including the engineering of host resistance to aphids into commercial wheat varieties, are required. Previous studies have identified ancestor wheat, *Triticum monococcum* accessions MDR045 and MDR049, with resistance against the grain aphid, *Sitobion avenae*. To test the hypothesis that resistance can be accounted for by antixenosis (reduced attractiveness of host plants) via the release of repellent volatile organic compounds (VOCs), we explored the response of *S. avenae* to MDR045 and MDR049 following *S. avenae* herbivory, using behaviour and electrophysiology experiments.

**RESULTS:**

In four‐arm olfactometry assays, alate *S. avenae* showed aphid density‐dependent reduced preference to VOC extracts from *T. monococcum* MDR045 and MDR049. By contrast, alate *S. avenae* showed aphid density‐dependent increased preference to extracts from aphid‐susceptible hexaploid wheat, *Triticum aestivum* var. Solstice and *T. monococcum* MDR037. Coupled gas chromatography‐electroantennography (GC‐EAG), using the antennae of alate *S. avenae*, located 24 electrophysiologically active compounds across all tested accessions. Synthetic blends created from 21 identified EAG‐active compounds confirmed bioactivity of corresponding VOC extracts in four‐arm olfactometry assays against alate *S. avenae*.

**CONCLUSION:**

Our data suggest that resistance of *T. monococcum* MDR045 and MDR049 to *S. avenae* can be at least partially accounted for by antixenosis through antennal perception of specific repellent VOC blends induced by *S. avenae* feeding behaviour. © 2024 The Author(s). *Pest Management Science* published by John Wiley & Sons Ltd on behalf of Society of Chemical Industry.

## Graphical abstract

A blend of 21 volatile organic compounds (VOCs) released from *Sitobion avenae‐*infested *Triticum monococcum* accessions MDR045 and MDR049 was identified and shown to induce antixenosis towards alate *S. avenae.*

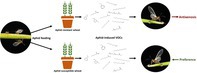

## 1 INTRODUCTION

Wheat, *Triticum aestivum* L. (Poaceae), is a major global food crop, which has consistently been in the top five most‐produced commodities worldwide between 2015 and 2021, forming part of the staple diet of a large portion of the global population Food and Agriculture Organisation of the United Nations (FAO) (https://www.fao.org/faostat). Within the United Kingdom (UK), wheat had the highest yield production compared with all other crops in 2021, of 14.5 million tonnes valued at £2.7 billion, showcasing its importance as a food crop within the UK FAO (https://www.fao.org/faostat). Aphids (Hemiptera: Aphididae) are major agricultural pests on wheat and other cereals, causing significant damage by reducing nutrient and assimilate availability via phloem feeding, by viral transmission, and by reducing photosynthesis due to aphid honeydew enabling saprophytic fungal growth on leaves.^1^, ^2^ These combined factors, particularly the viruses that aphids transmit, make aphids the most economically damaging cereal pests, leading to yield losses of 5–80% in wheat.^3^

Aphid infestations have mainly been controlled through the use of insecticides.^4^ However, the increasing presence of insecticide resistance across aphid populations, the initial and re‐registration costs of insecticides and the banning of working insecticides due to their environmental impact is reducing the options farmers have to control these pests.^4^, ^5^, ^6^ This has led to new initiatives to tackle aphid infestations, one of them being investigations into naturally occurring aphid resistance across cereal lines, which can potentially be engineered into commercial wheat varieties.^7^ This approach has been successful against the Russian wheat aphid *Diuraphis noxia* Kurdjumov, with several resistance genes identified and integrated into modern wheat.^8^ However, suitable resistance traits remain lacking for the two major aphid cereal pests in the UK and northern Europe, the English grain aphid, *Sitobion avenae* F. and the bird‐cherry‐oat aphid, *Rhopalosiphum padi* L., both of which are vectors for barley yellow dwarf virus (BYDV).^9^

Although aphid resistance has been identified against both *S. avenae* and *R. padi* in *T. aestivum* varieties,^10^, ^11^, ^12^ ancient wheat varieties have been found to be more resistant.^13^, ^14^, ^15^ This was observed in settling assays, where fewer *R. padi* settled on 10 of 12 tested *Triticum boeticum* Boiss. and *Triticum monococcum* L. varieties compared with *T. aestivum*, indicating that volatile organic compounds (VOCs) play a role in reduced aphid settlement.^16^ Further studies identified two promising *T. monococcum* varieties, MDR045 and MDR049, that show signs of aphid resistance against both *S. avenae* and *R. padi*.^17^, ^18^, ^19^, ^20^ *Triticum monococcum* is a diploid ancestor wheat closely related to the A genome donor of hexaploid wheat, *Triticum urartu* Thumanjan ex. Gandilyan.^21^ In field studies, both MDR045 and MDR049 had lower aphid numbers and MDR049 had more aphid predators than *T. aestivum*.^18^ Laboratory studies further showed that VOC extracts from *R. padi*‐infested and un‐infested MDR049 reduced preference of *R. padi* alate in four‐arm olfactometry assays, with six compounds identified to be potentially involved in this activity.^22^ Overall, these studies suggest antixenosis (reduced attractiveness) plays a role in the *S. avenae* and *R. padi* resistance mechanisms of MDR045 and MDR049.

In the context of aphid–plant interactions, intrinsically produced plant VOCs act as cues for aphid alightment on hosts (attractant or antixenotic), while herbivore‐induced plant volatiles can act as antixenotics or arrestants to aphids, attractants to aphid natural enemies or priming agents for neighbouring plants.^23^, ^24^ The use of VOCs in integrated pest management (IPM) strategies is a well‐established practice. They can be synthetic or natural and derived from either insects, such as pheromones, or plants, and are used in IPM strategies to catch or disorientate pests, recruit natural enemies or prime neighbouring plants for enhanced defence responses.^25^, ^26^, ^27^ Furthermore, VOCs, presented either *in planta* or as synthetic blends, can be incorporated into push–pull IPM strategies, reducing the need for insecticides.^27^, ^28^ Alternatively, identification of the genetic mechanisms involved in plant VOC‐mediated aphid resistance facilitates their incorporation into modern crop varieties via genetic engineering or classical breeding approaches.^3^, ^9^, ^29^ Genetic approaches reduce the need for pest protection products and allow for the development of a crop variety with multiple aphid resistance mechanisms, reducing the chances of the pest overcoming this resistance. This approach has been trialled with limited success through the incorporation of synthetic genes into wheat, rice (*Oryza sativa* L.) and cotton (*Gossypium hirsutum* L.). However, the approach still holds promise for the incorporation of genes from more closely related species.^30^, ^31^, ^32^

Following the identification of aphid resistance in *T. monococcum* MDR045 and MDR049, we aimed to characterise the VOC‐mediated aphid resistance mechanisms within these two germplasms. VOC extracts from *S. avenae*‐infested MDR045 and MDR049 were screened, alongside VOCs from aphid‐infested MDR037 and *T. aestivum* Solstice (aphid‐susceptible), in behavioural (four‐arm olfactometer) assays with alate *S. avenae*, to assess whether bioactivity is aphid‐density dependent. Coupled gas chromatography–electroantennography (GC–EAG) and coupled GC–mass spectrometry (GC–MS) were used to locate and identify bioactive VOCs within extracts.

## 2 MATERIALS AND METHODS

### 2.1 Insect and plant material

*Sitobion avenae* originating from the Rothamsted farm (Hertfordshire, UK), coordinates: 51° 48′ 36.8″ N, 0° 22′ 34.4″ W, were reared on wheat, *T. aestivum* Solstice (Advanta Seeds UK Ltd), in ventilated Perspex cages kept at 20 °C, 60–70% humidity and at a 16 h:8 h light/dark regime. Plants were replaced every 2 weeks to maintain an apterous aphid culture. Alates were developed when needed by allowing the culture to become overcrowded. Aphids were retrieved from cultures using a fine‐haired brush and placed in clip cages when required for use.

*Triticum monococcum* MDR037, MDR045, and MDR049 seeds were provided by the Wheat Genetic Improvement Network (WGIN) and *T. aestivum* Solstice seeds were provided by Rothamsted Research; seeds were stored at 4 °C until use. Seeds were sown in Rothamsted Prescription Mix (Petersfield Products, Leicester, UK) and grown in controlled environment rooms at 21 °C and 16 h:8 h light/dark regime until required for use. All experiments were run using 14‐day‐old plants.

### 2.2 Dynamic headspace collection (air entrainment)

Fourteen‐day‐old plants were subjected to 0, 1, 5, 10, and 25 aphid density treatments, using apterous *S. avenae* confined on the flag leaf of each plant in clip cages.^33^ Dynamic headspace collection was carried out using air entrainment kits (Pye volatile collection kit; Pye, Kings Walden, UK) after placement of clip cages on plants, with plants enclosed in transparent cooking bags (Sainsbury's Supermarkets Ltd, UK) secured by wire ties. Porapak Q adsorbent tubes consisting of a 4 mm internal diameter borosilicate tube filled with 50 mg Porapak Q (Supelco, Bellefonte, PA, USA) sandwiched between two glass wool plugs was used for the collection of headspace extracts. Charcoal‐filtered air was passed into the cooking bag at 500 mL min^−1^ and pulled out, through the adsorbent tube, at 400 mL min^−1^, collecting VOCs emitted by the plants on the adsorbent tubing over 24 h. Trapped VOCs were recovered from the Porapak Q tubes by eluting with redistilled diethyl ether (750 μL; Fisher Scientific, Loughborough, UK). Headspace extracts were concentrated to 100 μL under a gentle flow of nitrogen and stored at −20 °C until required for use. Four replicates were carried out for each treatment using an alpha design with additional Latinisation by blocks such that each treatment type would be present in every possible position of the experiment design across replicates. Following dynamic headspace collection, all plant shoots were collected, dried in an oven at 80 °C overnight and weighed.

### 2.3 Aphid behaviour assays

The bioactivity of VOC extracts against alate *S. avenae* was assessed using four‐arm olfactometer assays.^34^ The olfactometer consisted of three layers of 6 mm thick Perspex with an internal diameter of 115 mm, in which the middle layer was fashioned to consist of four side arms/areas at 90° angles to each other, and a central area. Each side arm narrowed at the perimeter leading to a 3 mm hole, in which glass syringes can be placed to test headspace extracts. The lower layer was lined with a 110 mm diameter Whatman type 1 filter paper (Maidstone, UK) providing traction for the walking aphid. The top Perspex layer contained a central hole (3 mm diameter), which was connected to a vacuum pump pulling air through each olfactometer arm at a rate of 75 mL min^−1^. Prior to use, glassware was washed with Teepol, rinsed with water and acetone and baked at 130 °C overnight, while the Perspex was washed with Teepol, rinsed with water and 70% ethanol and allowed to air‐dry. The olfactometer was placed in a box lined with black paper to reduce the effect of external stimuli and illuminated from above by diffuse uniform light from two 18 W/35 white, fluorescent light bulbs. Headspace extract samples from replicates 1 and 4 from each treatment (containing the most similar GC profiles across replicates) were combined and evaporated down to 100 μL under flow of nitrogen. Next, 10 μL of the pooled sampled, equivalent to the VOCs released by five plants over 20 min, was placed on a strip of Whatman type 1 filter paper and placed in the treatment glass syringe attached to one arm of the olfactometer. Furthermore, 10 μL of redistilled diethyl ether was used as a control in each of the remaining three arms of the olfactometer. A single alate *S. avenae* was placed in the central chamber of the olfactometer and left to acclimatise for 2 min, after which the experiment was run for 16 min, rotating the olfactometer by 90° every 4 min. The time the aphid spent in each olfactometer arm was measured using the OLFA software (Udine, Italy, 1995). Each experiment was run at 22 °C and repeated ten times for each treatment, replacing the aphid after each run. To assess repellent activity, the olfactometer assay method was adjusted to have three treatments arms and one control arm.

Synthetic blends of identified EAG‐active compounds were tested in four‐arm olfactometry assays to confirm whether they are responsible for the aphid behavioural responses induced by VOC extracts. A synthetic blend for each VOC extract that produced a significant behavioural response was made in a way that the applied dose in 10 μL solution mimicked that of the corresponding VOC extract. The concentration of VOC extracts and volumes used to create synthetic blends from authentic standards are shown in Supporting Information Table S1 and S2, respectively. Diethyl ether (10 μL) served as control as described earlier.

The following experiments were conducted, in which *n* represents the aphid density:

- *Triticum monococcum* MDR049 VOC extract (*S. avenae n* = 0, 1, 5, 10, 25) *versus* solvent control.
- *Triticum monococcum* MDR045 VOC extract (*S. avenae n* = 0, 1, 5, 10, 25) *versus* solvent control.
- *Triticum monococcum* MDR037 VOC extract (*S. avenae n* = 0, 1, 5, 10, 25) *versus* solvent control.
- *Triticum aestivum* Solstice VOC extract (*S. avenae n* = 0, 1, 5, 10, 25) *versus* solvent control.
- *Triticum monococcum* MDR049 synthetic blend (*S. avenae n* = 1, 5, 10, 25) *versus* solvent control.
- *Triticum monococcum* MDR045 synthetic blend (*S. avenae n* = 25) *versus* solvent control.
- *Triticum monococcum* MDR037 synthetic blend (*S. avenae n* = 10) *versus* solvent control.
- *Triticum aestivum* Solstice synthetic blend (*S. avenae n* = 10, 25) *versus* solvent control.
- *Triticum monococcum* MDR049 VOC extract (*S. avenae n* = 25) *versus* solvent control, repellent assay.

### 2.4 Coupled gas chromatography–electroantennography (GC–EAG) analysis

To locate compounds within behaviourally active extracts collected from the two extreme aphid density treatments (*S. avenae n* = 0 and 25), coupled GC–EAG analysis was performed using the antennae of alate *S. avenae*. The set up has been previously described.^35^ Alate *S. avenae* antennae were excised at the base segment of the antenna, followed by removing the extreme tip, and suspended between two glass electrodes filled with ringer solution (without glucose). The glass electrodes were attached to silver chloride electrode (Ag‐AgCl) wires in a way that the antennal tip was brought into contact with the recording electrode. Headspace extract (2 μL) was injected into an Agilent 6890A GC fitted with a non‐polar HP1 column (50 m length × 0.32 mm inner diameter × 0.52 μm film thickness, J&W Scientific, Folsom, CA, USA), using helium as the carrier gas and a 60 min run time starting at 30 °C for 2 min, followed by a rise of 5 °C min^−1^ until 250 °C. Signals from the aphid antenna were amplified (UN‐06, Ockenfels Syntech GmbH, Kirchzarten, Germany) and monitored simultaneously with the GC‐FID (flame ionisation detector) outputs using Syntech GC/EAD for Windows software (version 2.3, September 1997). GC peaks were deemed to be EAG active if a response was elicited in at least three replicate runs, with a minimum of six replicates run per treatment. To determine and confirm behavioural activity of EAG‐active compounds, synthetic blends were tested in four‐arm olfactometry assays as described earlier.

### 2.5 Coupled GC–mass spectrometry (GC–MS) analysis

The Kováts index was calculated and coupled GC–MS analysis was performed for the tentative identification of electrophysiologically active peaks, using the same representative samples tested in GC–EAG analysis. VOC extracts (4 μL) were analysed on an Agilent 5977B GC‐MSD fitted with a non‐polar HP1 column (50 m length × 0.32 mm inner diameter × 0.52 μm film thickness; J&W Scientific), using the following conditions: 30 °C for 5 min, rising 5 °C min^−1^ to 150 °C followed by a 10 °C min^−1^ rise to 230 °C for a total run time of 60 min. Ionisation was by electron impact (70 eV, 220 °C). Tentative identification of compounds was achieved by comparison of spectra with the National Institute of Standards and Technology (NIST) mass spectral library (2020, NIST, Gaithersburg, MD, USA). Identification of compounds was confirmed by GC peak enhancement via co‐injection with authentic standards,^36^ using an Agilent 6890A GC fitted with a non‐polar HP1 column (50 m length × 0.32 mm inner diameter × 0.52 μm film thickness; J&W Scientific) starting at an oven temperature of 30 °C for 5 min, rising 5 °C min^−1^ to 150 °C, followed by a 10 °C min^−1^ rise to 230 °C for a total run time of 60 min. Quantification of electrophysiologically active compounds was determined from GC and GC–MS datasets using known amounts of alkane standards and dried shoot weights (Table S1).^37^

### 2.6 Chemicals

Ethylbenzene (99%), cyclohexanone (99%), heptanal (95%), benzaldehyde (99%), hexanoic acid (98%), 6‐methyl‐5‐hepten‐2‐one (99%), nonanoic acid (96%), octanal (99%), nonanal (95%), undecane (97%), decanal (99%), undecanal (97%), 4‐ethylbenzoic acid (99%), tetradecane (99%), hexadecane (99%), and heptadecane (99%) were obtained from Sigma‐Aldrich (Gillingham, UK). Acetoxyacetone (97%) and 3‐ethylphenol (95%) were obtained from Thermo Fisher Scientific (Hemel Hempstead, UK), 3‐ethylbenzaldehyde (95%) obtained from Flourochem Ltd (Hadfield, UK) and pentadecane (99%) obtained from Koch‐Light Laboratories Ltd (Colnbrook, UK).

(*E*)‐*β*‐Farnesene was synthesised in‐house using a modified route as previously reported.^38^ To a solution of (*E,E*)‐farnesol (1 g, 4.48 mmol) and 3,4‐dihydropyran (1.88 g, 22.40 mmol) in dichloromethane (DCM; 30 mL), under nitrogen, was added *p*‐toluenesulphonic acid (82 mg, 0.45 mmol) and the reaction stirred for 60 min. The reaction mixture was diluted with DCM before being washed with water, saturated sodium hydrogen carbonate (NaHCO_3_), dried using magnesium sulphate (MgSO_4_) and concentrated under vacuum. The crude product was purified on silica gel (4% diethyl ether in petroleum ether) to give 2‐(((2*E*,6*E*)‐3,7,11‐trimethyldodeca‐2,6,10‐trien‐1‐yl)oxy)tetrahydro‐2*H*‐pyran (1.00 g, 73% yield) as a colourless oil. To a solution of 2‐(((2*E*,6*E*)‐3,7,11‐trimethyldodeca‐2,6,10‐trien‐1‐yl)oxy)tetrahydro‐2*H*‐pyran (500 mg, 1.63 mmol) and 18‐crown‐6 (87 mg, 0.33 mmol) in tetrahydrofuran (THF; 25 mL), under nitrogen, was added potassium *t*‐butoxide (1.83 g, 16.30 mmol) and the reaction mixture heated to 65 °C for 8 h. The reaction mixture was poured into water and extracted with petroleum ether. The combined organics were dried (MgSO_4_) and concentrated under vacuum. The crude product was purified on silica gel (100% petroleum ether) to give (*E*)‐7,11‐dimethyl‐3‐methylenedodeca‐1,6,10‐triene ((*E*)‐*β*‐farnesene; 232 mg, 70% yield) as a colourless oil. All spectroscopic data were consistent with previously reported literature values.

### 2.7 Statistical analysis

Statistical analysis of olfactometry data was carried out by comparing mean time alate aphids spend between treatment and control arms (fixed effect) using a linear mixed model (LMM) in GenStat (2016, 18th Edition, VSN International Ltd, Hemel Hempstead, UK), accounting for random effects caused by replicates and olfactometer area. Data were visualised using R.^39^

For the statistical comparison of the concentrations of EAG active compounds between treatments, a dataset showing the standardised amount (ng g^−1^ leaf weight h^−1^) for the identified EAG‐active compounds in each VOC extract sample across all treatments was created. Standardisation was carried out using the collected dried shoot weights and GC peak areas from an alkane C7–C22 standard (100 ng each). Using R, the dataset was log2 transformed and comparison between treatments was carried out via principal component analysis (PCA) and adonis permutational multivariate analysis test using the packages ‘vegan’, ‘ropls’ and ‘factoextra’.^40^, ^41^, ^42^

## 3 RESULTS

### 3.1 Olfactometry assays

**Figure 1:**
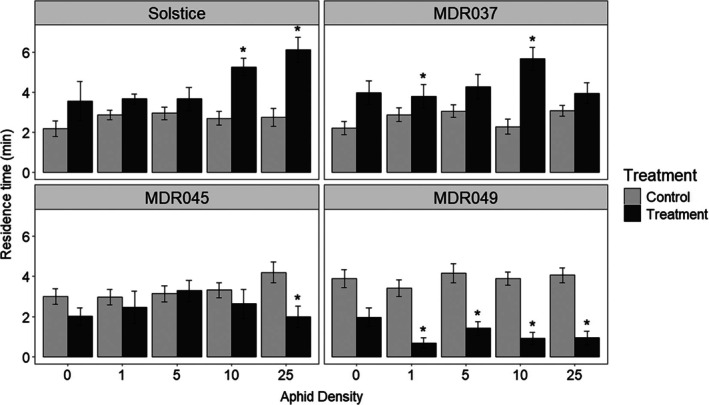
Behavioural response of alate grain aphids, *Sitobion avenae*, in a four‐arm olfactometer to VOCs collected for 24 h from *Triticum aestivum* var. Solstice and *Triticum monococcum* MDR037, MDR045, and MDR049 plants. VOCs were collected from plants with different aphid densities (*n* = 0, 1, 5, 10, and 25 per plant). Data are presented as the mean (min ± standard error) residence time spent in treatment and control olfactometer arms. The control in all experiments was diethyl ether. Asterisks indicate a significant difference between treatment and control (LMM test: *P* < 0.05).

Alate (winged) *S. avenae* spent less time (*P* < 0.05) in the arms containing the VOCs collected over a 24 h period from *S. avenae* (*n* = 1, 5, 10, and 25)‐damaged *T. monococcum* MDR049 compared with the solvent control (Fig. 1). However, alate *S. avenae* spent less time (*P* < 0.05) in the arms containing the VOCs collected over a 24 h period from *S. avenae* (*n* = 25)‐damaged *T. monococcum* MDR045, that is, only the highest aphid density, compared with the solvent control (Fig. 1). Alate *S. avenae* spent more time (*P* < 0.05) in the arms containing the VOCs from *S. avenae* (*n* = 10, 25)‐damaged *T. aestivum* var. Solstice and VOCs from *S. avenae* (*n* = 1, 10)‐damaged *T. monococcum* MDR037 compared with the solvent controls (Fig. 1).

Alate *S. avenae* spent less time (mean time = 1.25 min ± 0.27, *P* < 0.001) in the arms containing the VOCs collected over a 24 h period from *S. avenae* (*n* = 25)‐damaged *T. monococcum* MDR049 compared with the solvent control (mean time = 5.49 min ± 0.69) in an olfactometer repellence test (Supporting Information Fig. S1).

### 3.2 Electrophysiology and GC–MS analysis

**Figure 2:**
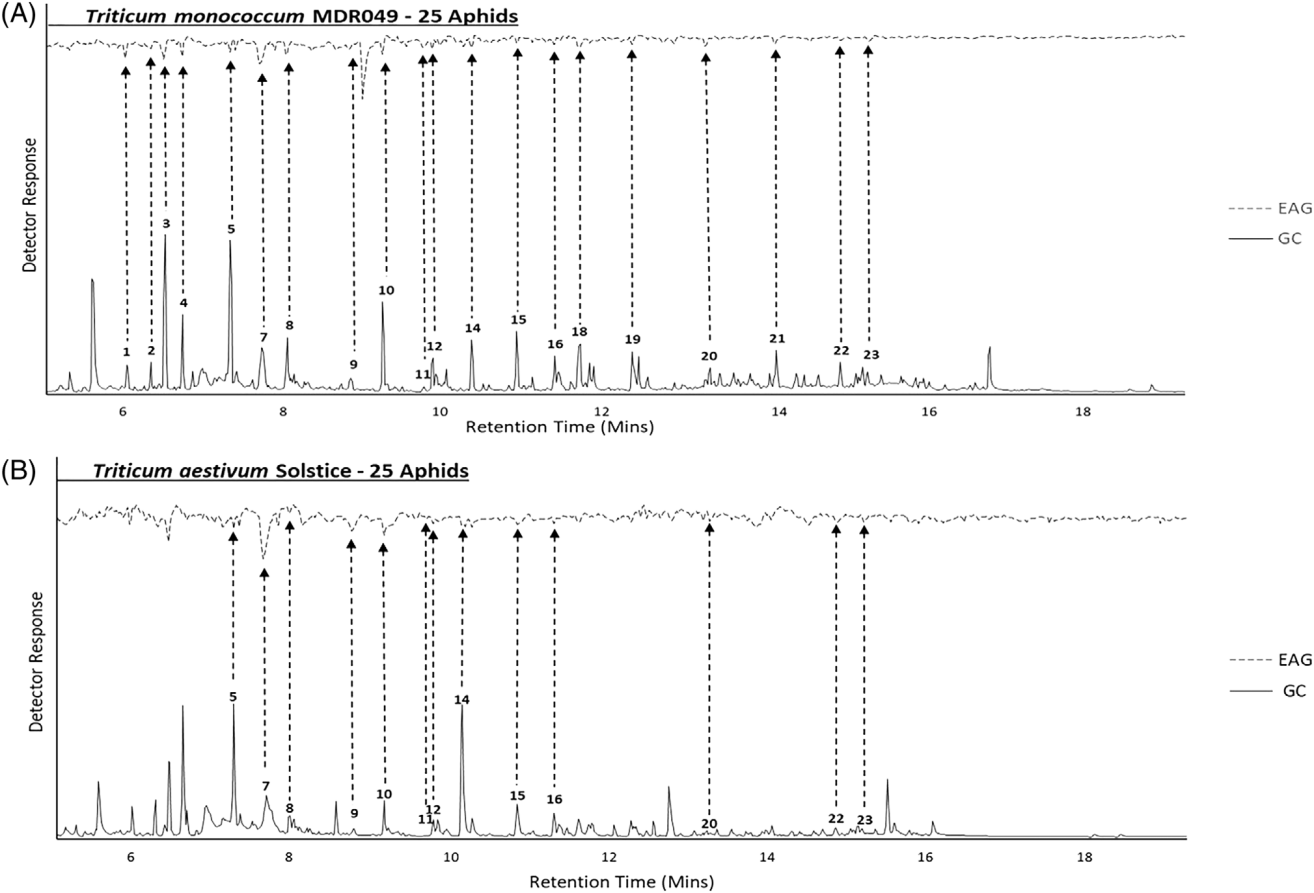
Representative coupled GC–EAG traces showing antennal responses of alate grain aphids, *Sitobion avenae* against headspace extracts collected from *S. avenae* (*n* = 25)‐infested *Triticum monococcum* MDR049 (A) and *T. aestivum* Solstice (B). Upper trace, response of antenna; lower trace, FID response. GC peak numbers correspond to compounds listed in Table 1 with arrows indicating their respective EAG peak. Identifications confirmed by GC peak enhancement using authentic standards.

Coupled GC–EAG analysis using the antennae of alate *S. avenae* located 24 electrophysiologically active compounds (Table 1) from VOC extracts collected from *T. monococcum* MDR037, MDR045, MDR049, and *T. aestivum* Solstice under 0 and 25 *S. avenae* infestation densities (Figs 2 and S2). Coupled GC–MS analysis and GC peak enhancement with authentic standards identified 21 of the 24 electrophysiologically active compounds as acetoxyacetone, ethylbenzene, cyclohexanone, heptanal, benzaldehyde, hexanoic acid, 6‐methyl‐5‐hepten‐2‐one, octanal, nonanal, undecane, 3‐ethylbenzaldehyde, 3‐ethylphenol, decanal, nonanoic acid, undecanal, 4‐ethylbenzoic acid, tetradecane, (*E*)‐*β*‐farnesene, pentadecane, hexadecane and heptadecane (Table 1). 3,4‐Dimethylbenzaldehyde, 2‐isopropyl‐5‐methyl‐1‐heptanol and 1‐ethyldecylbenzene identity could not be confirmed as authentic standards were not commercially available at the time of experiments.

**Table 1:** Electrophysiologically active compounds against *Sitobion avenae* alate antennae in headspace extracts of *Triticum monoccocum* MDR049, MDR045, MDR037, and *Triticum aestivum* Solstice collected under 0 and 25 *S. avenae* infestation densities, showing their tentative identification, assigned compound number, retention index (Kováts index) and ID confirmation by gas chromatography (GC) co‐injection

Tentative compound identification^†^	Compound number	Retention index^‡^	ID confirmed by co‐injection^§^
Acetoxyacetone	1	832	✓
Ethylbenzene	2	852	✓
Cyclohexanone	3	866	✓
Heptanal	4	882	✓
Benzaldehyde	5	930	✓
Hexanoic acid	6	955	✓
6‐Methyl‐5‐hepten‐2‐one	7	961	✓
Octanal	8	984	✓
Nonanal	9	1086	✓
Undecane	10	1100	✓
3‐Ethylbenzaldehyde	11	1133	✓
3‐Ethylphenol	12	1143	✓
3,4‐Dimethylbenzaldehyde	13	1159	✖
Decanal	14	1189	✓
Nonanoic acid	15	1245	✓
Undecanal	16	1281	✓
4‐Ethylbenzoic acid	17	1317	✓
2‐Isopropyl‐5‐methyl‐1‐heptanol	18	1325	✖
Tetradecane	19	1392	✓
(E)‐β‐Farnesene	20	1450	✓
Pentadecane	21	1496	✓
Hexadecane	22	1599	✓
Heptadecane	23	1703	✓
1‐Ethyldecylbenzene	24	1748	✖

^†^ Tentative identification of compounds was achieved by gas chromatography coupled with mass spectrometry (GC–MS) using the NIST spectral library.

^‡^ Using an HP‐1 non‐polar GC column.

^§^ ✓ = ID confirmed, ✖ = ID not confirmed.

PCA and multivariate analysis showed no significant differences in the VOC extract composition of the 21 identified electrophysiologically active compounds between *T. monococcum* MDR037, MDR045, MDR049, and *T. aestivum* var. Solstice across *S. avenae* density treatments (*n* = 0, 1, 5, 10, 25) (Fig. S3).

### 3.3 Behavioural activity of synthetic blends

**Figure 3:**
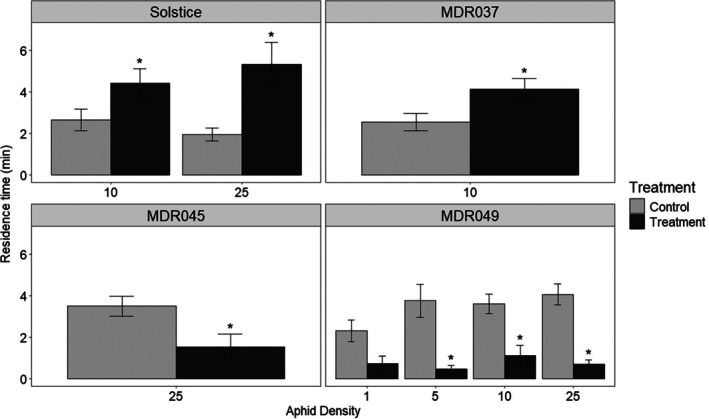
Behavioural response of alate grain aphids, *Sitobion avenae*, in a four‐arm olfactometer to synthetic blends of electrophysiologically active compounds identified from *Triticum aestivum* var. Solstice and *Triticum monococcum* MDR037, MDR045, and MDR049 plants, which induced a significant behavioural response (Fig. 1). Data are presented as the mean (min ± standard error) residence time spent in treatment and control olfactometer arms. The control in all treatments was diethyl ether. Asterisks indicate a significant difference between treatment and control (LMM test: *P* < 0.05).

In four‐arm olfactometer bioassays, alate *S. avenae* spent less time (*P* < 0.05) in the arms containing synthetic blends of electrophysiologically active compounds identified from the VOC extracts of MDR045 and MDR049 compared with solvent controls (Fig. 3), when presented at a dose equivalent to an *n* = 25 and *n* = 5, 10, 25 aphid density for MDR045 and MDR049, respectively. Alate *S. avenae* spent more time (*P* < 0.05) in the arms containing synthetic blends of electrophysiologically active compounds identified from the VOC extracts of *T. aestivum* var. Solstice and MDR037 compared with solvent controls (Fig. 3), when presented at a dose equivalent to an *n* = 10, 25 and *n* = 10 aphid density for Solstice and MDR037, respectively.

## 4 DISCUSSION

Our study provides an understanding of the aphid antixenotic mechanisms of *T. monococcum* MDR045 and MDR049, both of which have previously shown reduced *R. padi* and *S. avenae* numbers in the field.^18^ In olfactometry assays, VOC extracts from MDR049 induced significant reduced preference in alate *S. avenae*, compared with controls, starting from an *S. avenae* density of *n* = 1. In MDR045, VOC extracts induced significant reduced preference in alate *S. avenae*, compared with controls, from an *S. avenae* density of *n* = 25. This suggests that MDR049 has a threshold of one aphid required to induce VOC‐related resistance mechanisms, with MDR045 having a 25‐aphid threshold to induce a similar resistance mechanism. This reduced aphid preference is observed to be aphid species‐specific, previous work showing a significantly reduced *R. padi* preference compared with controls in olfactometry assays by MDR049 VOC extracts collected under a 50 *R. padi* infestation density (showing similar results to those observed here against *S. avenae*).^22^ However, MDR045 VOC extracts collected under a 50 *R. padi* infestation density induced no change in preference against *R. padi* compared with controls,^22^ contrasting the reduced preference from aphid‐infested MDR045 VOC extracts against *S. avenae* shown here. This species‐specific response has been previously observed in wheat, where VOC extracts from primed plants induced different responses in *R. padi* and *S. avenae* in olfactometry assays,^43^ highlighting the need to test against multiple aphid species when screening for aphid resistance in crops.

Similar reduced preference of *S. avenae* has been observed in *T. monococcum* however, the cultivar tested was not provided by the authors.^44^ Contrastingly, choice assays have identified the Brazilian cultivar *T. aestivum* BRS Timbauva, bred for BYDV resistance, as being disruptive to *R. padi* behaviour.^11^ Headspace extracts from uninfested *T. aestivum* Ciko induced a preferential response in *R. padi* ^45^; however, under aphid infestation, headspace extracts became repellent.^46^ This variability is not limited to wheat but is also observed in *Zea mays* (L.).^47^ A screening study on 132 Ethiopian and Chinese *T. aestivum* accessions, which included choice assays, identified three accessions: Lunxuan 266, 243726 and 213312 with antixenosis‐based resistance mechanisms against *Sitobion miscanthi* Takahashi.^48^ These studies highlight that VOC‐mediated aphid resistance mechanisms are plant genotype‐dependent and can vary highly between cultivars, explaining the variability in headspace extract bioactivity across *T. monococcum* MDR lines. Interestingly, less variability is observed across Triticale accessions for aphid preference, with choice assays showing similarly reduced preference of *R. padi* between *T. aestivum* Arapahoe and five tested Triticale accessions.^49^

MDR049 VOC extracts induced a significant reduced preference to alate *S. avenae* at a one *S. avenae* density, with MDR045 VOC extracts inducing this response at a 25 *S. avenae* density, compared with controls. The results showed a lack of a gradual reduction in *S. avenae* preference with increasing aphid densities within the two accessions. MDR049 VOC extracts collected at a zero‐aphid density already induced low preference towards alate *S. avenae*, compared with controls; therefore, a gradual decrease in *S. avenae* preference was unlikely since the initial preference was already low and indicates a level of intrinsically reduced preference (statistically non‐significant) towards *S. avenae* in MDR049. In fact, aphid densities higher than 25 aphids, that is, at 30, 70, and 100 aphid densities, induce distinct VOC composition changes at each density treatment in *Arabidposis thaliana* ((L.) Heynh.) under *Myzus persicae* (Sulzer) feeding.^50^ Furthermore, the induction of the phytohormones salicylic acid and jasmonic acid, which are both involved in aphid resistance,^51^ is shown to be aphid density‐dependent in *Medicago truncatula* (Gaertn.).^52^ In the case of *T. monococcum*, aphid density was shown to be positively correlated with presence of aphid predators on MDR045 and MDR049 in the field,^18^ which was also observed on *Rubus idaeus* (L.).^53^ The current study shows that the aphid threshold required to induce resistance is genotype‐dependent. Aphid density‐dependent effects have also been demonstrated to be species‐specific, that is, the aphid density required to induce significant yield losses in winter wheat was a minimum of ten aphids per plant for *R. padi* and *D. noxia*, and 15 aphids per plant for *Schizaphis graminum* (Rondani).^54^

No unique EAG‐active compounds were identified in either the resistant or susceptible accessions, indicating the ratio of these compounds is the key factor in inducing the different behaviroual responses observed in the olfactometry assays. Interestingly, several compounds detected as EAG‐active in headspace extracts from certain accessions did not elicit a response in others, despite being present in the VOC blend. It has been shown that EAG responses are dose‐dependent, so the concentration of EAG‐active VOCs in headspace extracts where they did not elicit a response may have been too low to be detected by the aphid antennae.^55^ No significant differences were observed in the composition of headspace extracts between accessions when electrophysiologically active compounds were considered. This indicates that statistically non‐significant but biologically important changes in VOC ratios are responsible for the contrasting behavioural activity induced by the aphid‐susceptible (Solstice and MDR037) and resistant (MDR045 and MDR049) accessions. This was confirmed by testing synthetic blends of the identified electrophysiologically active VOCs at their natural doses, which induced the same behavioural response as their corresponding headspace extracts. Considering the minor differences in headspace extract composition between susceptible and resistant lines, identifying key compounds and their doses responsible for the activity of the repellent accessions is difficult. Future work is required to assess synthetic blend activity via omitting single constituents to determine the key compounds responsible for headspace extract activity of MDR045 and MDR049. Additionally, extract bioactivity should be confirmed in the future under field conditions.

The importance of compound ratios in VOC blend activity against aphids has been previously demonstrated.^56^ For example, for the black‐bean aphid, *Aphis fabae* (Scopoli), a blend of ten compounds from headspace extracts of its host plant were identified as being responsible for eliciting behavioural preference, while the individual compounds themselves induced an antixenosis response.^57^, ^58^, ^59^ Similarly, a synthetic blend of 32:1 (*E*)‐2‐hexenal/(*E*)‐caryophyllene that simulated the natural proportions found in *Humulus lupulus* (L.) headspace attracted *Phorodon humuli* (Schrank); however, this preference was lost at a 1:1 ratio.^60^ It has been suggested that blends of active VOCs are perceived as distinct odours and therefore when in contact with individual components of the blend or with blends at different ratios, the odour becomes unrecognisable to the insect, explaining this change in behavioural response.^61^ Due to plant–plant variation, VOC blends of the same genotype may differ when grown under the same conditions,^62^ and it has been proposed that a major compound in a VOC blend can be replaced by other compounds to elicit the same response, accounting for this natural variation.^56^ It was not determined in the current study which compounds are crucial for the observed behavioural activity (antixenosis via repellent compounds and preference by attractants); thus, further work is required to test synthetic blends in olfactometry assays omitting single compounds to check if the observed responses change. Furthermore, this study assessed VOC extracts collected from 14‐day old plants, but it is unknown whether antixenosis is maintained at different stages of plant growth in MDR045 and MDR049. VOC profiles have been shown to change across different plant growth stages,^63^, ^64^ which in turn could affect VOC bioactivity. Whether or not MDR045 and MDR049 maintain antixenosis at different plant growth stages should be investigated in the future.

Of the 21 identified EAG‐active compounds, 12 are green leaf volatiles (GLVs) and related compounds: heptanal, octanal, nonanal, decanal, undecanal, hexanoic acid, nonanoic acid, undecane, tetradecane, pentadecane, hexadecane, and heptadecane. GLVs are common plant VOCs known to be involved in biotic resistance responses against both herbivory and pathogen infection.^65^, ^66^, ^67^, ^68^ This is also the case for benzenoids,^45^, ^57^, ^69^ forming the second largest class of active compounds identified in *T. monococcum* headspace extracts, including ethylbenzene, benzaldehyde, 3‐ethylbenzaldehyde, 3‐ethylphenol, 3,4‐dimethylbenzaldehyde, 4‐ethylbenzoic acid, and 1‐ethyldecylbenzene. Many of the identified compounds have been documented to be physiologically active against aphids and involved in both attractive/preferential and repellent/antixenotic responses, depending on their ratios in blends. Heptanal, nonanal, decanal, benzaldehyde, tetradecane, hexadecane, heptadecane, and 4‐ethylbenzaldehyde were identified as active compounds, using olfactometry assays, in headspace extracts from wheat and oat, inducing preference in *R. padi* alate.^45^ When tested individually, only heptanal, nonanal, decanal and benzaldehyde induced preference. Similarly, octanal, decanal, undecanal, (*E*)‐*β*‐farnesene, benzaldehyde and 6‐methyl‐5‐hepten‐2‐one were identified as physiologically active compounds from field bean *Vicia faba* L., headspace extracts that induced preference in *Aphis fabae*.^57^ When tested individually, octanal, decanal, undecanal, and (*E*)‐*β*‐farnesene induced an antixenotic response, while benzaldehyde, undecanal, and 6‐methyl‐5‐hepten‐2‐one were inactive; however, preference could be induced when they were combined in their naturally occurring ratios.^58^

The VOCs identified in this study also play a role in tri‐trophic cereal–aphid–virus interactions. Nonanal, decanal and undecane are present in headspace extracts from BYDV viruliferous and non‐viruliferous *T. aestivum* Lambert, with the individual compounds and BYDV‐infected headspace extracts eliciting a higher preference in *R. padi* in immigration bioassays, compared with the solvent control.^70^ Decanal concentrations were shown to be important in the VOC activity of BYDV‐viruliferous and non‐viruliferous *T. aestivum* Aikang‐58, with viruliferous *Schizaphis graminum* preferring non‐viruliferous plants with lower decanal concentrations, while non‐viruliferous aphids preferred viruliferous plants with higher decanal concentrations.^71^ Nonanoic acid, nonanal, (*E*)‐*β*‐farnesene, ethylbenzene, cyclohexanone and benzaldehyde are involved in aphid predator attraction.^72^, ^73^, ^74^ The presence of these compounds in headspace extracts of MDR045 and MDR049, and field observations of higher aphid predator presence in MDR049,^18^ may indicate that aphid predator attraction is involved in aphid resistance mechanisms of these accessions; however, this will need further laboratory testing for confirmation.

## 5 CONCLUSION

In conclusion, the results in this study suggest a role for VOCs in aphid antixenosis displayed by *T. monococcum* MDR045 and MDR049. The induced VOCs appear to be present across the aphid‐resistant and susceptible accessions, implying that VOC blend composition is crucial for conveying the antixenosis effect. Furthermore, these results provide a platform for the incorporation of VOC‐based aphid resistance into modern elite wheat varieties, which can then be deployed in IPM strategies to reduce the direct and indirect negative impacts of aphid infestations, thereby reducing dependence on insecticides. Further work is required to elucidate the functional genes involved in the production of VOCs in MDR045 and MDR049 and the mechanisms involved in their regulation.

## CONFLICT OF INTEREST STATEMENT

The authors declare there are no conflict of interests.

## Supporting information


**Table S1.** Mean normalised amount (ng g^−1^ leaf weight h^−1^) of EAG‐active compounds emitted by *Triticum aestivum* Solstice and *Triticum monococcum* MDR037, MDR045 and MDR049 plants subjected to alate *Sitobion avenae* densities (*n* = 0, 1, 5, 10, 25).
**Table S2.** Volume (μL) of authentic standard solutions (1 mg mL^−1^ in diethyl ether) required to create a 10× concentration synthetic blend in diethyl ether (1 mL) for the EAG‐active compounds from *Triticum aestivum* Solstice and *Triticum monococcum* MDR037, MDR045 and MDR049 headspace extract. Synthetic blends were subsequently diluted for use in olfactometry assays.
**Figure S1.** Behavioural response of alate grain aphids, *Sitobion avenae*, in a four‐arm olfactometer to VOCs collected for a period of 24 h from *S. avenae* (*n* = 25)‐infested *Triticum monococcum* MDR049 plants. Data are presented as the mean (min ± SE) time spent in treatment and control olfactometer arms. The experiment included three treatment arms and one control arm. The control was diethyl ether. Asterisks indicate a significant difference between treatment and control (ANOVA: *P* < 0.05).
**Figure S2.** Representative coupled GC‐EAG traces showing antennal responses of alate grain aphids, *Sitobion avenae*, against headspace extracts collected from *S. avenae* (*n* = 0)‐infested *Triticum monococcum* MDR049 and *Triticum aestivum* Solstice and *S. avenae* (*n* = 0, 25)‐infested *T. monococcum* MDR037 and MDR045 for 24 h. Upper trace, response of antenna; lower trace, FID response. GC peak numbers correspond to compounds listed in Table 1 with arrows indicating their respective EAG peak. Identifications confirmed by GC peak enhancement using authentic standards.
**Figure S3.** Principal component analysis (PCA) comparing EAG active VOC composition between *Sitobion avenae* (*n* = 0, 1, 5, 10, 25)‐infested *Triticum monococcum* MDR037, MDR045, MDR049 and *Triticum aestivum*. Ellipses represent the 95% confidence limits at each accession. Adonis permutation analysis provided *P* > 0.05 across all treatment combinations.

## Data Availability

The data that support the findings of this study are available from the corresponding author upon reasonable request.
