# Supplementary material for: Characterisation of aphid antixenosis in aphid‐resistant ancestor wheat, Triticum monococcum
Source: Pest Manag Sci. 2024 Aug 17;81(11):7321–9. doi: 10.1002/ps.8380 (PMC12539387; doi:10.1002/ps.8380)
Supplement: Supplementary file 1 — Table S1. Mean normalised amount (ng g−1 leaf weight h−1) of EAG‐active compounds emitted by Triticum aestivum Solstice and Triticum monococcum MDR037, MDR045 and MDR049 plants subjected to alate Sitobion avenae densities (n = 0, 1, 5, 10, 25). Table S2. Volume (μL) of authentic standard solutions (1 mg mL−1 in diethyl ether) required to create a 10× concentration synthetic blend in diethyl ether (1 mL) for the EAG‐active compounds from Triticum aestivum Solstice and Triticum monococcum MDR037, MDR045 and MDR049 headspace extract. Synthetic blends were subsequently diluted for use in olfactometry assays. Figure S1. Behavioural response of alate grain aphids, Sitobion avenae, in a four‐arm olfactometer to VOCs collected for a period of 24 h from S. avenae (n = 25)‐infested Triticum monococcum MDR049 plants. Data are presented as the mean (min ± SE) time spent in treatment and control olfactometer arms. The experiment included three treatment arms and one control arm. The control was diethyl ether. Asterisks indicate a significant difference between treatment and control (ANOVA: P < 0.05). Figure S2. Representative coupled GC‐EAG traces showing antennal responses of alate grain aphids, Sitobion avenae, against headspace extracts collected from S. avenae (n = 0)‐infested Triticum monococcum MDR049 and Triticum aestivum Solstice and S. avenae (n = 0, 25)‐infested T. monococcum MDR037 and MDR045 for 24 h. Upper trace, response of antenna; lower trace, FID response. GC peak numbers correspond to compounds listed in Table 1 with arrows indicating their respective EAG peak. Identifications confirmed by GC peak enhancement using authentic standards. Figure S3. Principal component analysis (PCA) comparing EAG active VOC composition between Sitobion avenae (n = 0, 1, 5, 10, 25)‐infested Triticum monococcum MDR037, MDR045, MDR049 and Triticum aestivum. Ellipses represent the 95% confidence limits at each accession. Adonis permutation analysis provided P > 0.05 across all treatm [file PS-81-7321-s001.docx]

Supplementary material

**Table S1**. Mean normalised amount (ng g^-1^ leaf weight hr^-1^) of EAG-active compounds emitted by *Triticum aestivum* Solstice and *T. monococcum* MDR037, MDR045 and MDR049 plants subjected to alate *Sitobion avenae* densities (n = 0, 1, 5, 10, 25).

| Accession | Aphid Density | Acetoxy acetone | Ethylbenzene | Cyclohexanone | Heptanal | Benzaldehyde | Hexanoic Acid | 6-Methyl-5-hepten-2-one | Octanal | Nonanal | Undecane | 3-EthylBenzaldehyde | 3-EthylPhenol | Decanal | Nonanoic Acid | 4-EthylBenzoic Acid | Tetradecane | (E)-β-Farnesene | Pentadecane | Hexadecane | Heptadecane |
| --- | --- | --- | --- | --- | --- | --- | --- | --- | --- | --- | --- | --- | --- | --- | --- | --- | --- | --- | --- | --- | --- |
| MDR049 | 0 | 3.2 | 9.4 | 35.3 | 28.8 | 7.3 | 2.4 | 0.7 | 6.7 | 9.2 | 0.4 | 2.2 | 0.3 | 5.7 | 0.2 | 0.4 | 3.9 | 0.4 | 0.5 | 1.8 | 1.4 |
| MDR049 | 1 | 5.9 | 14.7 | 44.0 | 39.3 | 11.7 | 3.7 | 0.9 | 6.6 | 12.6 | 1.6 | 1.2 | 0.0 | 7.8 | 2.6 | 0.2 | 4.1 | 0.2 | 0.5 | 1.5 | 0.6 |
| MDR049 | 5 | 5.3 | 14.5 | 31.5 | 34.6 | 8.4 | 2.9 | 0.3 | 7.9 | 13.1 | 0.4 | 0.9 | 0.2 | 7.5 | 0.6 | 0.4 | 3.8 | 0.2 | 1.3 | 1.5 | 0.9 |
| MDR049 | 10 | 2.2 | 4.3 | 14.5 | 14.6 | 2.7 | 0.6 | 0.2 | 2.6 | 3.9 | 0.1 | 0.4 | 0.0 | 1.6 | 0.1 | 0.0 | 1.2 | 0.2 | 0.1 | 0.8 | 0.6 |
| MDR049 | 25 | 3.8 | 9.8 | 29.7 | 23.2 | 7.4 | 1.8 | 0.0 | 6.4 | 8.8 | 0.4 | 2.8 | 0.0 | 4.0 | 0.8 | 0.1 | 2.9 | 0.6 | 0.2 | 2.0 | 1.5 |
| MDR045 | 0 | 5.2 | 31.9 | 26.1 | 51.8 | 18.3 | 7.5 | 1.6 | 17.3 | 21.8 | 1.1 | 1.4 | 1.6 | 13.5 | 0.6 | 0.9 | 7.8 | 0.1 | 0.1 | 2.8 | 1.6 |
| MDR045 | 1 | 3.2 | 10.5 | 21.6 | 24.3 | 6.1 | 1.8 | 0.3 | 5.4 | 9.9 | 0.4 | 1.4 | 0.3 | 5.7 | 0.4 | 0.4 | 4.0 | 0.6 | 1.7 | 2.2 | 1.6 |
| MDR045 | 5 | 2.2 | 6.1 | 16.5 | 14.8 | 3.2 | 0.7 | 0.2 | 2.4 | 4.6 | 0.4 | 1.3 | 0.0 | 1.7 | 0.7 | 0.0 | 1.4 | 0.2 | 0.1 | 1.2 | 0.6 |
| MDR045 | 10 | 5.7 | 14.7 | 30.6 | 37.9 | 8.9 | 2.4 | 1.1 | 8.4 | 11.3 | 0.5 | 0.8 | 0.3 | 6.2 | 0.9 | 0.3 | 2.9 | 0.1 | 0.5 | 2.2 | 0.7 |
| MDR045 | 25 | 2.8 | 7.5 | 30.1 | 22.2 | 6.1 | 1.0 | 0.3 | 5.3 | 9.5 | 0.3 | 1.1 | 0.0 | 4.2 | 0.0 | 0.3 | 3.2 | 1.2 | 0.4 | 1.2 | 0.9 |
| MDR037 | 0 | 3.3 | 21.1 | 51.7 | 45.9 | 13.0 | 4.5 | 0.1 | 11.8 | 16.2 | 0.7 | 1.4 | 0.2 | 10.2 | 0.7 | 0.3 | 5.1 | 0.4 | 0.3 | 3.4 | 1.7 |
| MDR037 | 1 | 3.0 | 17.5 | 19.9 | 34.4 | 12.1 | 5.6 | 2.5 | 13.9 | 18.3 | 1.0 | 1.6 | 0.7 | 10.4 | 0.8 | 0.3 | 5.8 | 0.2 | 0.0 | 1.6 | 0.5 |
| MDR037 | 5 | 4.2 | 19.4 | 21.9 | 41.3 | 13.8 | 5.0 | 0.4 | 12.7 | 16.5 | 0.8 | 1.7 | 0.9 | 9.3 | 0.8 | 0.4 | 5.5 | 0.5 | 0.0 | 2.8 | 1.7 |
| MDR037 | 10 | 6.3 | 15.2 | 33.5 | 45.1 | 9.0 | 2.0 | 1.8 | 8.9 | 13.0 | 0.6 | 1.0 | 0.2 | 7.6 | 0.6 | 0.7 | 4.8 | 0.4 | 0.7 | 1.9 | 1.2 |
| MDR037 | 25 | 3.5 | 7.9 | 18.6 | 23.4 | 5.6 | 1.6 | 0.0 | 5.7 | 8.4 | 0.2 | 0.6 | 0.1 | 3.9 | 0.0 | 0.2 | 1.1 | 1.7 | 0.1 | 0.9 | 1.0 |
| Solstice | 0 | 1.3 | 3.1 | 8.9 | 9.0 | 2.5 | 0.4 | 0.1 | 2.2 | 2.7 | 0.1 | 0.5 | 0.0 | 1.2 | 0.1 | 0.1 | 0.2 | 0.0 | 0.1 | 0.5 | 0.2 |
| Solstice | 1 | 4.4 | 30.8 | 9.5 | 56.7 | 12.8 | 6.7 | 4.8 | 14.1 | 19.9 | 1.6 | 1.7 | 0.0 | 13.2 | 0.9 | 1.1 | 10.0 | 0.4 | 0.0 | 4.9 | 3.5 |
| Solstice | 5 | 3.7 | 10.0 | 8.5 | 30.2 | 5.6 | 1.2 | 1.3 | 5.1 | 6.8 | 0.4 | 0.8 | 0.0 | 3.9 | 0.2 | 0.3 | 2.6 | 0.3 | 0.4 | 1.5 | 0.9 |
| Solstice | 10 | 2.1 | 4.9 | 11.2 | 15.5 | 6.2 | 2.0 | 0.1 | 6.0 | 10.0 | 0.4 | 1.8 | 0.5 | 6.0 | 0.7 | 3.0 | 3.8 | 0.2 | 0.3 | 1.7 | 0.7 |
| Solstice | 25 | 5.4 | 11.3 | 24.8 | 47.0 | 9.1 | 2.0 | 0.1 | 8.2 | 10.8 | 0.5 | 1.2 | 0.1 | 6.6 | 0.3 | 0.3 | 4.1 | 2.1 | 0.2 | 1.3 | 0.8 |

| Accession | MDR049 | MDR049 | MDR049 | MDR049 | MDR045 | MDR037 | Solstice | Solstice |
| --- | --- | --- | --- | --- | --- | --- | --- | --- |
| Aphid Density | *1* | *5* | *10* | *25* | *25* | *10* | *10* | *25* |
| Acetoxyacetone | 4.1 | 3.4 | 1.8 | 1.8 | 1.8 | 4.8 | 1.4 | 4.1 |
| Ethylbenzene | 10.1 | 9.1 | 3.7 | 4.4 | 3.7 | 11.5 | 2.8 | 8.3 |
| Cyclohexanone | 30 | 21.1 | 12.5 | 13.6 | 17.9 | 26.1 | 8.3 | 17.1 |
| Heptanal | 7.8 | 5.2 | 2.6 | 3.5 | 3.6 | 6.8 | 3.9 | 7.2 |
| Benzaldehyde | 28 | 23.3 | 12.3 | 11.4 | 13.7 | 35.6 | 11.5 | 35.1 |
| Hexanoic Acid | 2.7 | 1.8 | 0.6 | 0.9 | 0.7 | 1.4 | 1.4 | 1.7 |
| 6-Methyl-5-hepten-2-one | 0.5 | 0.2 | 0.2 | 0 | 0.2 | 1.3 | 0.1 | 0.2 |
| Octanal | 4.8 | 5.1 | 2.5 | 3.1 | 3.3 | 7 | 4.1 | 7 |
| Nonanal | 10.4 | 10 | 4.2 | 4.9 | 6.8 | 12.1 | 8.6 | 10.9 |
| Undecane | 1.2 | 0.3 | 0.2 | 0.2 | 0.2 | 0.5 | 0.3 | 0.5 |
| 3-Ethylbenzaldehyde | 0.9 | 0.7 | 0.6 | 1.5 | 0.9 | 1 | 1.6 | 1.2 |
| 3-Ethylphenol | 0 | 0.2 | 0 | 0 | 0 | 0.2 | 0.4 | 0 |
| Decanal | 6.4 | 5.5 | 1.8 | 2.2 | 3 | 6.6 | 4.9 | 6.3 |
| Nonanoic Acid | 2 | 0.5 | 0.1 | 0.4 | 0 | 0.5 | 0.8 | 0.3 |
| 4-Ethylbenzoic Acid | 0.2 | 0.3 | 0 | 0.1 | 0.2 | 0.5 | 4.5 | 0.2 |
| Tetradecane | 2.9 | 2.4 | 1.1 | 1.4 | 1.9 | 3.9 | 2.4 | 3.2 |
| (E)-β-Farnesene | 0.1 | 0.1 | 0.2 | 0.3 | 0.8 | 0.3 | 0.1 | 1.7 |
| Pentadecane | 0.4 | 0.8 | 0.1 | 0.1 | 0.3 | 0.6 | 0.1 | 0.2 |
| Hexadecane | 1 | 1.1 | 0.7 | 1 | 0.8 | 1.6 | 1.5 | 1.1 |
| Heptadecane | 0.4 | 0.6 | 0.5 | 0.7 | 0.6 | 1 | 0.5 | 0.7 |

**Table S2.** Volume (µl) of authentic standard solutions (1mg/ml in diethyl ether) required to create a 10x concentration synthetic blend in diethyl ether (1 mL) for the EAG-active compounds from Triticum aestivum Solstice and T. monococcum MDR037, MDR045 and MDR049 headspace extract. Synthetic blends were subsequently diluted for use in olfactometry assays.


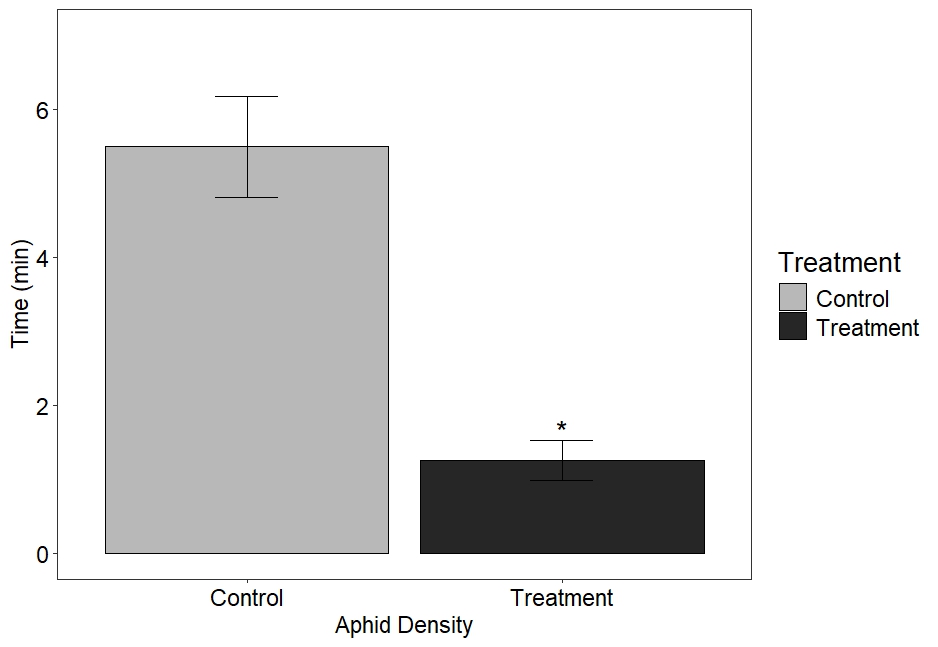


**Figure S1** Behavioural response of alate grain aphids, Sitobion avenae, in a four-arm olfactometer to VOCs collected for a period of 24h from S. avenae (n=25)-infested Triticum monococcum MDR049 plants. Data are presented as the mean (min ±SE) time spent in treatment and control olfactometer arms. The experiment included three treatment arms and one control arm. The control was diethyl ether. Asterisks indicate a significant difference between treatment and control (ANOVA: P<0.05).


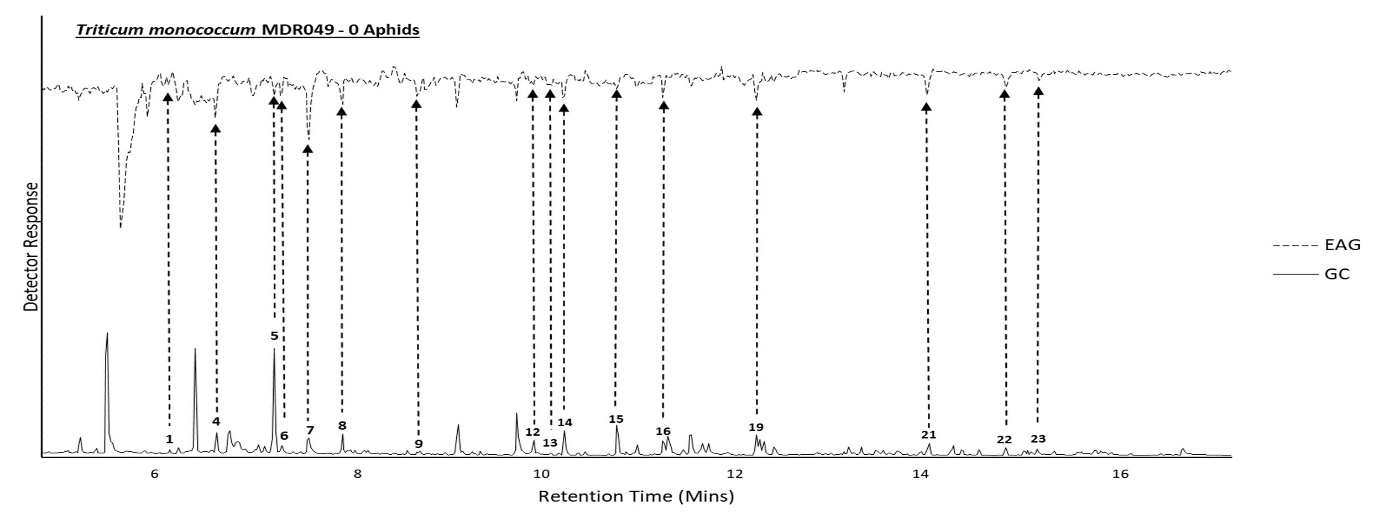

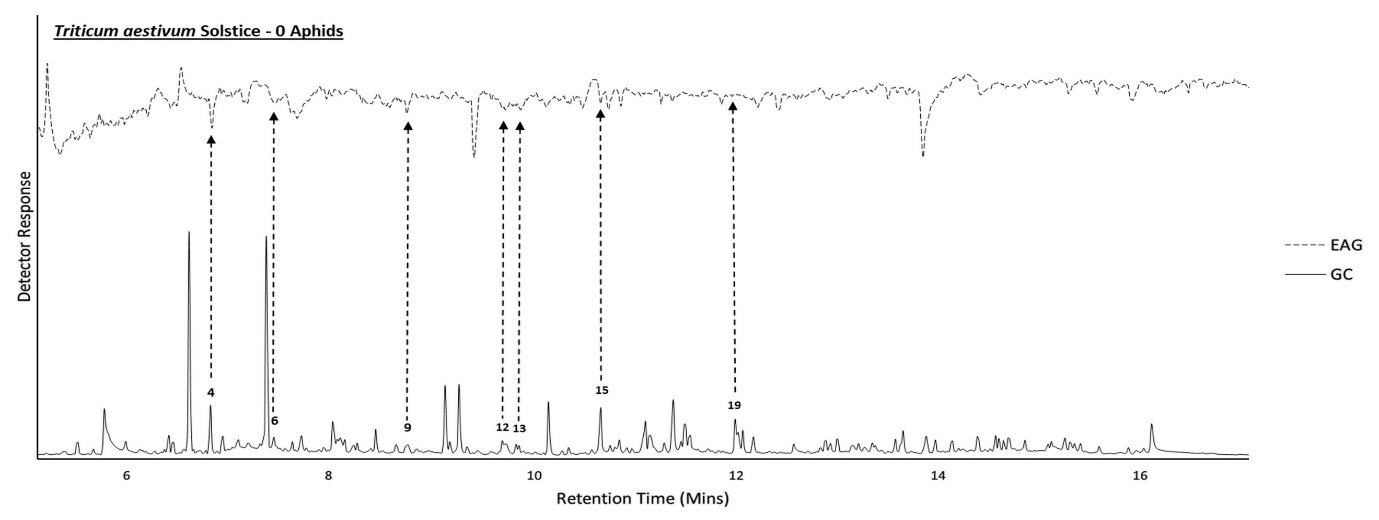

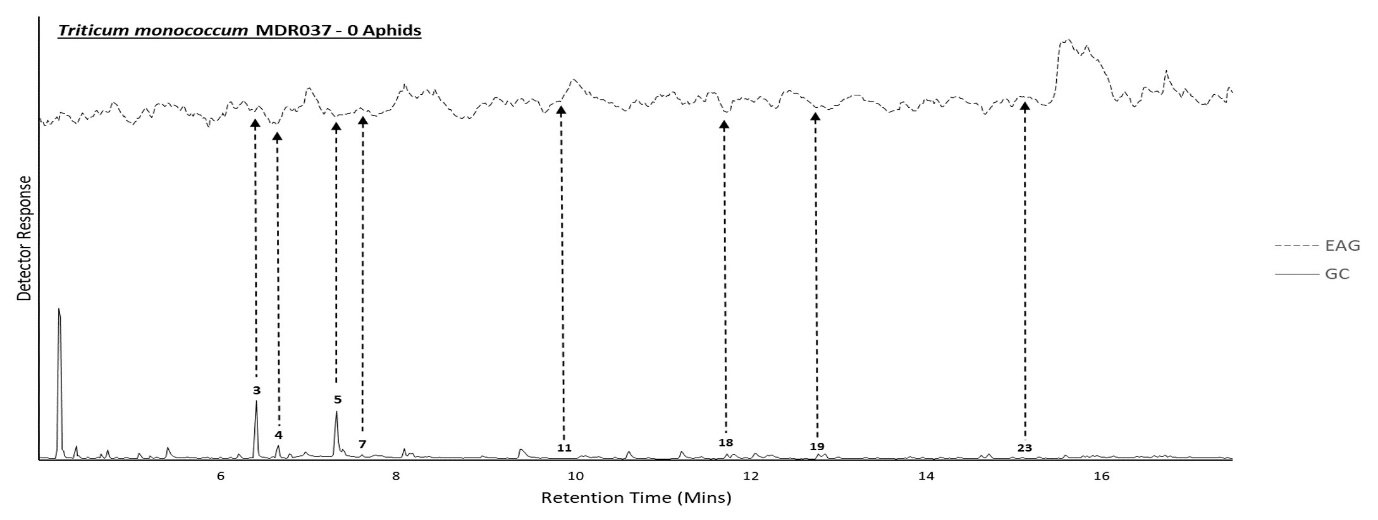

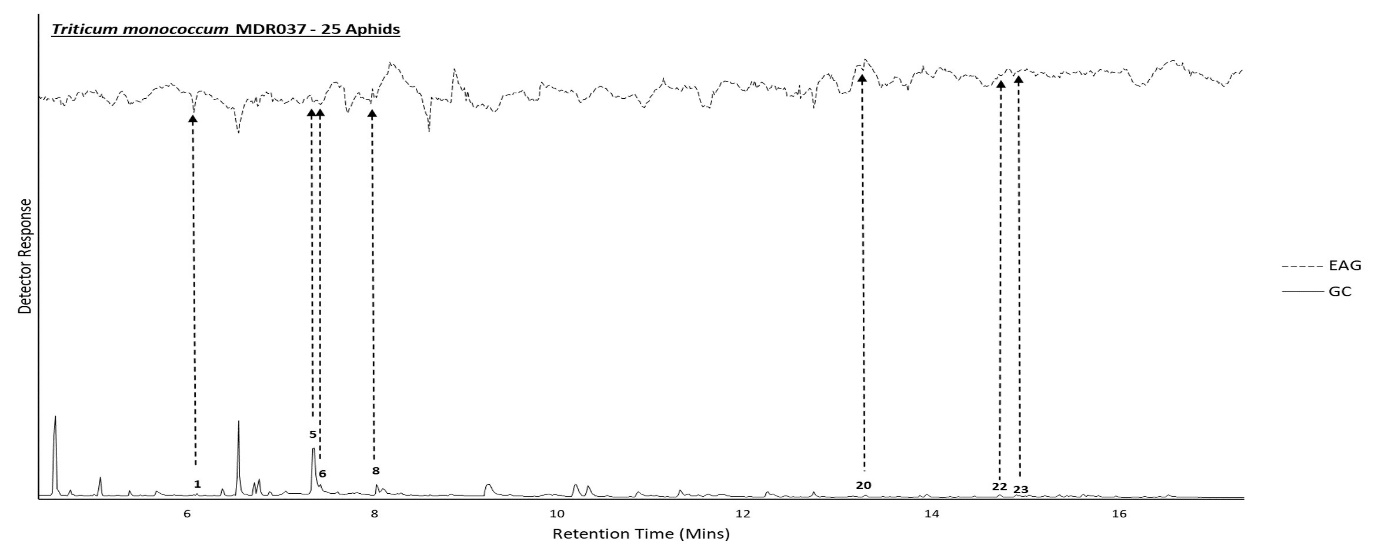


**Figure S2** Representative coupled GC-EAG traces showing antennal responses of alate grain aphids, Sitobion avenae, against headspace extracts collected from S. avenae (n=0)-infested T. monococcum MDR049 and T. aestivum Solstice and S. avenae (n=0, 25)-infested T. monococcum MDR037 and MDR045 for 24 hr. Upper trace, response of antenna; lower trace, FID response. GC peak numbers correspond to compounds listed in Table 1 with arrows indicating their respective EAG peak. Identifications confirmed by GC peak enhancement using authentic standards.


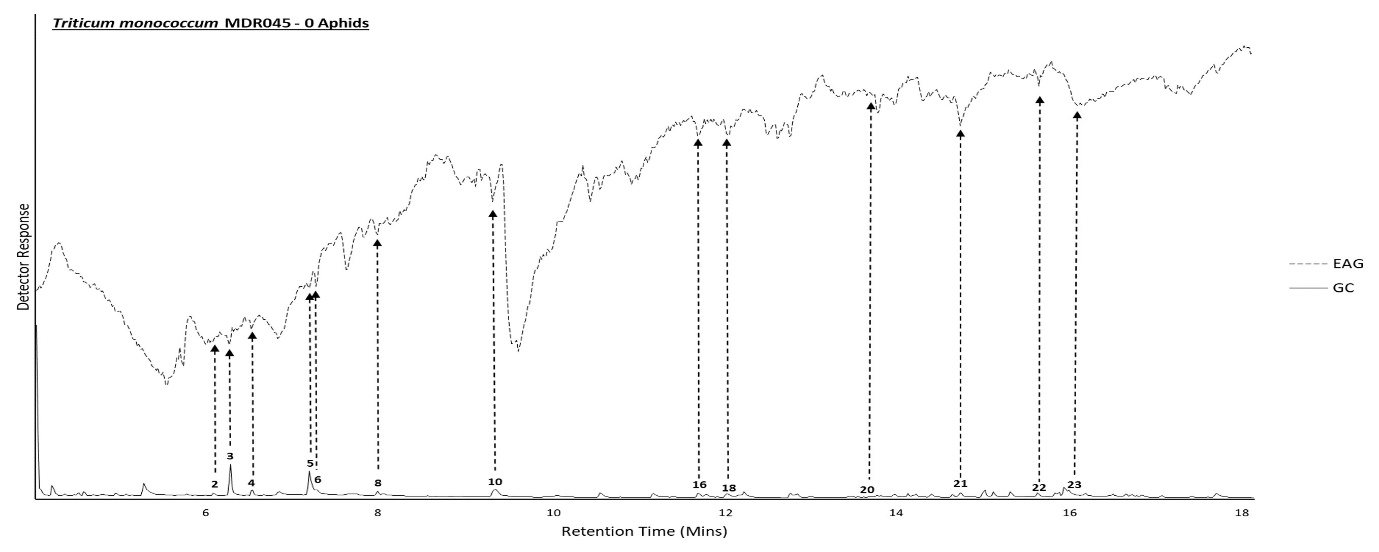

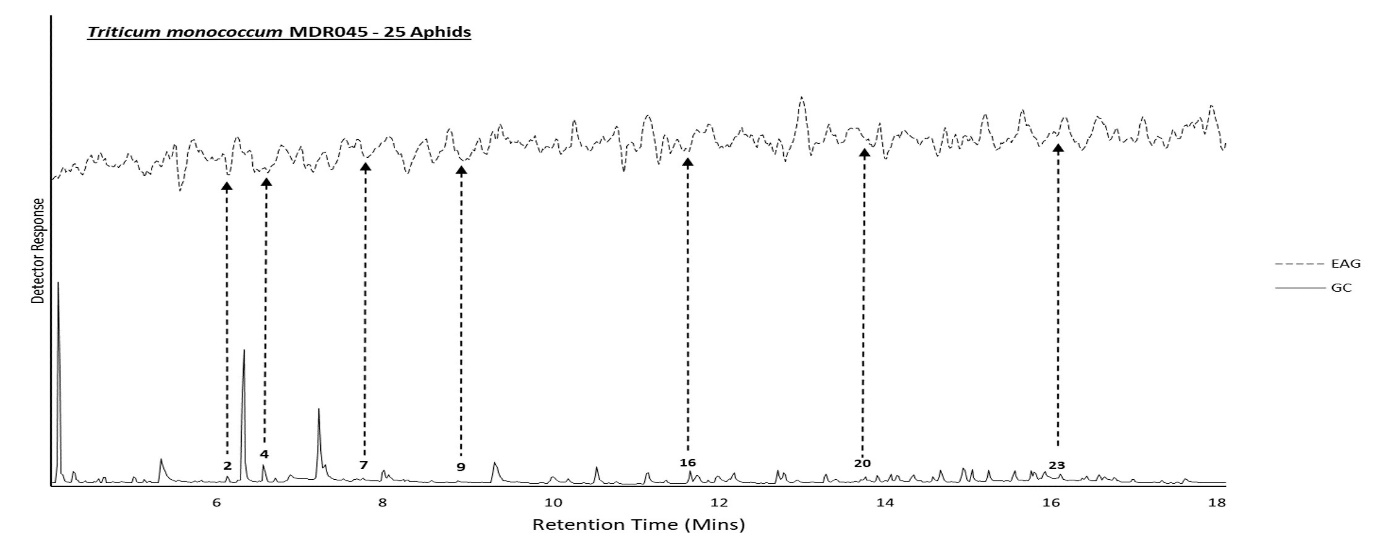

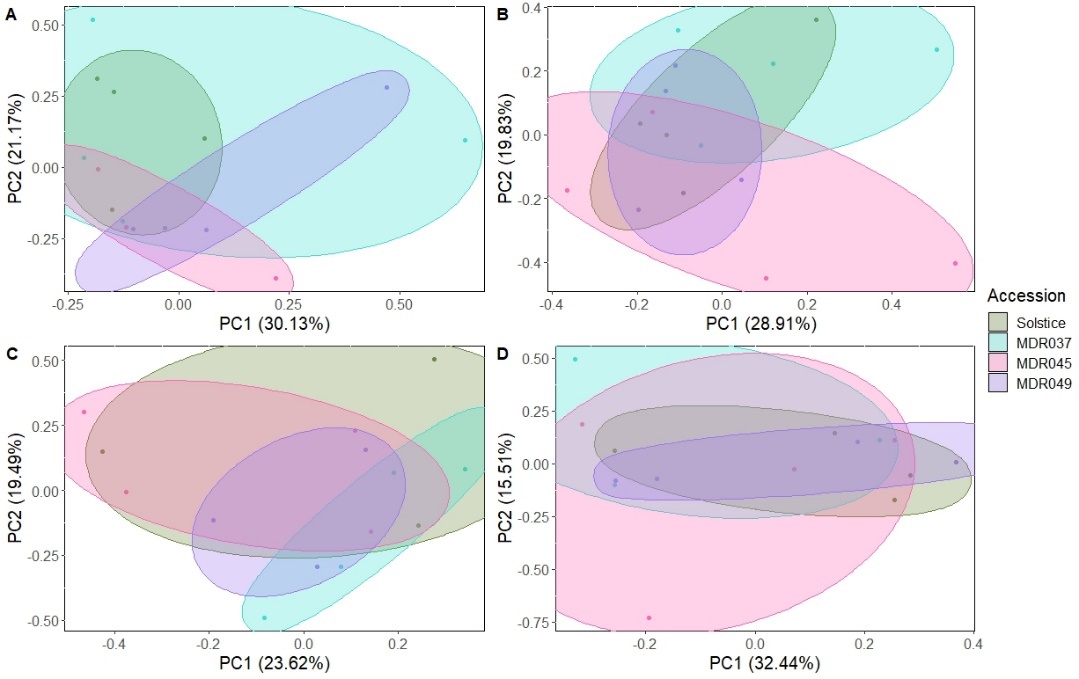


**Figure S3.** Principal component analysis (PCA) comparing EAG active VOC composition between S. avenae (n=0, 1, 5, 10, 25)-infested T. monoccocum MDR037, MDR045, MDR049 and T. aestivum. Ellipses represent the 95% confidence limits at each accession. Adonis permutation analysis provided P > 0.05 across all treatment combinations.
